# Reduction of multi-compartmental biophysical models by incremental, automated retuning of their parameters and synaptic weights

**DOI:** 10.1186/1471-2202-15-S1-P178

**Published:** 2014-07-21

**Authors:** Thomas G Close, Ben Torben-Nielsen, Erik De Schutter

**Affiliations:** 1 Computational Neuroscience Unit, Okinawa Institute of Science and Technology, Okinawa, Japan; 2 University of Antwerp, Antwerp, Belgium

## 

When simulating large networks of neuronal models with conductance-based membrane dynamics, neuronal models with large numbers of compartments are often not practical due to the computational resources required. In this case, a common approach is to seek a model with fewer compartments that approximates the behaviour of the full compartmental model [1,2]. While analytical techniques to reduce multi-compartmental models consisting of passive, quasi-active or restricted classes of active models exist [3-5], in general, the reduction of active models requires parameter retuning in order to retain functionally relevant properties of the model. We propose an incremental, automated parameter retuning approach to reduce the number of compartments, which avoids labor-intensive manual retuning and ensures key functional properties of the model are maintained.

Starting from the full compartmental model, the number of compartments is incrementally reduced by merging compartment subsets into single, electronically equivalent compartments. After each compartment reduction phase, all parameters of the model, including synaptic weights, are locally optimised to best match the dynamics and post-synaptic potentials of the full compartmental model using multiple objectives, including phase-plane trajectory histograms of membrane voltage and internal states [6,7]. The successive local optimisations during the incremental reduction in the number of compartments ensures the model parameters do not transition to qualitatively distinct regions of the parameter space. The multi-compartmental models are iteratively reduced in this manner until key functional properties can no longer be maintained within specified tolerances.

The proposed algorithm is demonstrated on multi-compartmental models of neurons in the cerebellum.
